# Vitamin E Attenuates Glyphosate-Induced Multi-Organ Toxicity and Redox Imbalance in Rats: Biochemical and Histopathological Evidence

**DOI:** 10.3390/vetsci13080740

**Published:** 2026-07-25

**Authors:** Muhammet Can, Cihangir Işık, Cengiz Gökbulut, Şehver Ege Hïsmïoğullari, Gülay Turan, Akın Usta, Özgür Bulmuş, Mustafa Hilmi Yaranoğlu

**Affiliations:** 1 Department of Forensic Medicine, Faculty of Medicine, Balıkesir University, 10145 Balıkesir, Türkiye; 2 Department of Forensic Medicine, Balıkesir Atatürk City Hospital, 10100 Balıkesir, Türkiye; 3 Department of Pharmacology, Faculty of Medicine, Balıkesir University, 10145 Balıkesir, Türkiye; 4 Department of Pharmacology and Toxicology, Faculty of Veterinary Medicine, Balıkesir University, 10145 Balıkesir, Türkiye; 5 Department of Pathology, Faculty of Medicine, Balıkesir University, 10145 Balıkesir, Türkiye; 6 Department of Gynaecology and Obstetrics, Faculty of Medicine, Balıkesir University, 10145 Balıkesir, Türkiye; 7 Department of Physiology, Faculty of Medicine, Balıkesir University, 10145 Balıkesir, Türkiye; 8 Experimental Animal Production, Care and Research Centre, Balıkesir University, 10145 Balıkesir, Türkiye

**Keywords:** α-tocopherol, herbicide toxicity, hepatoprotection, antioxidant supplementation, wistar rats, redox homeostasis

## Abstract

Glyphosate, the world’s most widely used herbicide, is of growing concern in both human and veterinary medicine because livestock, companion, and working animals may be chronically exposed through feed, water, and grazing on treated land. This study evaluates the protective effects of vitamin E (*α*-tocopherol acetate) against chronic glyphosate-induced hepatotoxicity, multi-organ histopathological damage, and disturbances of systemic redox balance in male Wistar rats. Fifty-six rats were allocated to six groups: control, low-dose glyphosate (LG, 560 mg/kg/day), high-dose glyphosate (HG, 1120 mg/kg/day), LG + vitamin E, HG + vitamin E, and vitamin E alone. Treatments were administered by oral gavage for 30 days at equalised dosing volumes. Serum oxidant/antioxidant markers—total oxidant status (TOS), total antioxidant status (TAS) and the oxidative stress index (OSI)—hepatic enzymes (AST, ALT, ALP, LDH), renal markers (urea, creatinine) and cardiac marker creatine kinase-MB (CK-MB) were measured; liver, lung, kidney, testis and heart were examined histopathologically. Glyphosate produced dose-dependent hepatocellular enzyme elevations (AST, ALT) and progressive multi-organ histopathological injury. Contrary to expectation, serum TOS and OSI did not increase in the glyphosate-only groups but were instead lower than in controls, accompanied by a compensatory rise in TAS—indicating that circulating oxidant markers may underestimate tissue-level oxidative injury at this exposure duration. Vitamin E co-administration markedly enhanced TAS, lowered TOS relative to the corresponding glyphosate group (HG + VitE vs. HG, *p* = 0.007), reduced OSI and substantially attenuated histopathological damage across all examined organs. Vitamin E thus mitigates glyphosate-induced tissue injury and improves systemic antioxidant capacity, supporting its potential as a protective dietary antioxidant in animals subject to chronic glyphosate exposure.

## Simple Summary

Glyphosate is the most widely used weed killer in the world. Farm animals, companion animals, and working animals can be exposed to it over long periods through feed, water, and grazing on treated pasture, which makes its safety a genuine veterinary concern. In this study, rats—a standard model for mammalian toxicity—received glyphosate for 30 days, and some were also given vitamin E, a natural antioxidant present in many feedstuffs. Glyphosate damaged several organs, including the liver, lungs, kidneys, testes, and heart. Although the blood markers of oxidant load did not rise as expected, the animals’ antioxidant defences increased and the tissue damage itself was consistent with oxidative injury. Co-treatment with vitamin E strengthened antioxidant capacity and protected the organs from much of the damage. These findings suggest that vitamin E may help limit the harmful effects of long-term glyphosate exposure in animals and could serve as a simple, protective dietary supplement.

## 1. Introduction

Glyphosate (N-(phosphonomethyl)glycine) is a broad-spectrum systemic herbicide that accounts for approximately 40% of global pesticide use, making it the most widely applied active ingredient worldwide [1,2]. In Turkey alone, it is registered in 2664 commercial formulations, reflecting its deep integration into contemporary agricultural practice [3]. The global glyphosate market is projected to expand at a compound annual growth rate of 9.5%, reaching an estimated USD 17.11 billion by 2034 [4]. Because glyphosate is applied so extensively on croplands and pastures, domestic and production animals—cattle, sheep, and horses grazing sprayed forage, as well as dogs and other companion animals on farms—represent an important and frequently overlooked route of chronic mammalian exposure, giving the question direct veterinary relevance [5,6].

Glyphosate exerts its herbicidal action by inhibiting 5-enolpyruvylshikimate-3-phosphate (EPSP) synthase, thereby blocking aromatic amino acid biosynthesis in plants. Although this pathway is absent in mammals, increasing epidemiological and experimental evidence raises concern about adverse effects in non-target species [5,6]. The International Agency for Research on Cancer (IARC) classified glyphosate as “probably carcinogenic” (Group 2A) in 2015, based on evidence for oxidative stress-mediated genotoxicity [7,8]. Toxicokinetic studies report 30–35% gastrointestinal absorption and peak plasma concentrations within 1–2 h [9,10]. Mechanistically, glyphosate’s chelating properties enable binding of essential minerals—including manganese, magnesium, selenium and iron—potentially contributing to micronutrient deficiencies and downstream chronic pathology [11,12]. At the cellular level, chronic glyphosate exposure promotes oxidative stress by increasing reactive oxygen species (ROS) and impairing endogenous antioxidant defences, with consequent lipid peroxidation and macromolecular damage [12,13].

Experimental studies have linked glyphosate and glyphosate-based formulations to injury in several specific organ systems. In the liver, chronic exposure elevates transaminases and induces hepatocellular degeneration, congestion, and periportal fibrotic change [11,12,14,15]. In the kidney, glomerular and tubular structural changes have been reported even when serum urea and creatinine remain within reference limits [12]. Reproductive studies describe impaired spermatogenesis and seminiferous-epithelium damage, consistent with the high polyunsaturated-fatty-acid content and lipid-peroxidation susceptibility of testicular tissue [13,16]. Effects on pulmonary and cardiac tissue are less well-characterised but have been attributed to systemic ROS generation following gastrointestinal absorption [17,18]. A common mechanistic thread across these organs is oxidative membrane damage, which provides a rational target for antioxidant intervention.

Vitamin E, comprising tocopherols and tocotrienols, is the principal lipid-soluble antioxidant in mammalian tissues; *α*-tocopherol is the most biologically potent isoform [19,20]. It protects polyunsaturated fatty acids in cell membranes from oxidative degradation by intercepting peroxyl radicals, thereby maintaining cellular redox homeostasis [20,21]. Beyond its basal physiological role, dietary *α*-tocopherol supplementation has repeatedly been shown to raise systemic antioxidant capacity and to mitigate chemically induced tissue injury in experimental models [21,22]. In veterinary practice, vitamin E is an established feed supplement: in ruminants, poultry, and companion animals, it improves antioxidant status, membrane stability, and reproductive and immune function, and it has protective effects against a range of oxidative and xenobiotic insults [19,22].

In earlier rodent toxicology studies, subchronic glyphosate doses in the 560–1120 mg/kg/day range reliably elicit measurable organ pathology within 30 days [16,23]. Systemic redox status can be assessed with the automated colorimetric TOS and ABTS-based TAS methods of Erel, from which the OSI is derived; these assays are validated, widely used measures of oxidant/antioxidant balance in rodent and human serum [24,25], and OSI (TOS/TAS × 10) is an established composite index of systemic oxidative burden [26,27].

The present study was designed to: (i) characterise the toxic effects of chronic glyphosate exposure on hepatic, renal, and cardiac biomarkers in male Wistar rats; (ii) assess associated histopathological changes across multiple organ systems with microscopic evidence; and (iii) investigate the protective efficacy of vitamin E supplementation against glyphosate-induced redox imbalance and organ dysfunction.

## 2. Materials and Methods

### 2.1. Ethical Approval and Animal Care

All animal experiments were conducted in accordance with the ARRIVE (Animal Research: Reporting of In Vivo Experiments) guidelines. The study was carried out in accordance with the working guidelines and related directives of the Animals (Scientific Procedures) Act 1986, EU Directive 2010/63/EU on the protection of animals used for scientific purposes, and the NIH Guidelines on the Care and Use of Laboratory Animals. The study received formal approval from the Balıkesir University Experimental Animal Ethics Committee (Reference: 2026/1-11; Date: 5 February 2026). Experiments were performed at the Balıkesir University Experimental Animal Research Centre. Male animals were used exclusively to eliminate confounding effects of sex-hormone variability on oxidative stress parameters.

### 2.2. Animals and Experimental Design

Fifty-six healthy male Wistar rats (8–10 weeks; body weight 200–250 g) were obtained from the institutional animal facility and maintained under standard conditions (12 h light/dark cycle, 22 ± 2 °C, 55 ± 5% humidity) with ad libitum access to standard chow and water. Following one-week acclimatisation, rats were randomly allocated to six groups with balanced mean body weights. The test substance was a commercial herbicide formulation containing 48% glyphosate isopropylamine salt (active ingredient: N-(phosphonomethyl)glycine); the stated glyphosate doses (560 and 1120 mg/kg/day) refer to the administered formulation. The glyphosate formulation was freshly diluted in distilled water and administered by oral gavage; *α*-tocopherol acetate was dissolved in olive oil (3 mL/kg), which was also given to the control and glyphosate-only groups as the vehicle, so that vehicle load and total gavage volume were identical across all six groups. Group allocations, doses, vehicle, and dosing volumes are summarised in Table 1.

### 2.3. Biochemical Analyses

At the conclusion of the 30-day period, animals were fasted overnight and deeply anaesthetised with isoflurane. Blood was collected by cardiac puncture under deep anaesthesia, after which the animals were euthanised in accordance with the institutional ethical guidelines. Serum was obtained by centrifugation at 3000× *g* for 10 min and stored at −80 °C until analysis. Total Oxidant Status (TOS) was measured by an automated colorimetric method (μmol H_2_O_2_ Eq/L); Total Antioxidant Status (TAS) by the automated ABTS method (mmol Trolox Eq/L); and the Oxidative Stress Index (OSI) calculated as (TOS/TAS) × 10 [24,25]. Hepatic enzymes (AST, ALT, ALP, LDH), renal markers (urea, creatinine) and creatine kinase-MB (CK-MB) were measured using a fully automated analyser (Roche Cobas C501, Roche Diagnostics, Mannheim, Germany) with manufacturer-supplied reagent kits.

### 2.4. Histopathological Examination

Tissue samples from liver, lung, kidney, testis and heart were fixed in 10% neutral-buffered formalin, dehydrated, embedded in paraffin and sectioned at 4 μm. Brain and eye tissues were also collected and scored during the study; however, these organs are not reported in the present manuscript and their detailed evaluation is reserved for a separate report. Sections were stained with haematoxylin and eosin (H&E) and examined by two veterinary pathologists blinded to group allocation, each scoring independently with discrepancies resolved by consensus, using a light microscope (Olympus BX53, Olympus Corporation, Tokyo, Japan) at 100× and 200× magnification. Inflammation, fibrosis, congestion and degeneration were scored semi-quantitatively (0 = absent, 1 = mild, 2 = moderate, 3 = severe). Spermatogenesis was assessed using a modified Johnsen score (1–10). Because H&E alone cannot definitively confirm collagen deposition, renal and hepatic sections were additionally stained with Masson’s trichrome, which confirmed interstitial/portal collagen (fibrosis) in these organs [28]; collagen-specific staining was not available for the lung, so the pulmonary fibrosis score, based on H&E only, is reported as provisional (see Limitations). Group mean scores for every organ and lesion, together with the complete per-animal scoring data, are provided in Supplementary Materials, Table S1.

### 2.5. Statistical Analysis

Statistical analyses were performed in IBM SPSS Statistics v29.0 (IBM Corp., Armonk, NY, USA) [29]. Normality was assessed by the Shapiro–Wilk test. Because of non-normal distributions, continuous variables are expressed as median (minimum–maximum). The Kruskal–Wallis H test was used for multi-group comparisons, followed by Dunn’s post hoc test with Bonferroni correction. Effect sizes are reported as r = Z/√N. Significance was set at α = 0.05.

### 2.6. Use of Artificial Intelligence Tools

During the preparation of this manuscript, the authors used Claude (Anthropic) for the purposes of language editing and improving the English of the text. The authors have reviewed and edited the output and take full responsibility for the content of this publication.

## 3. Results

### 3.1. Biochemical Parameters

Biochemical and oxidative stress data are shown in Table 2.

For reference, the control and vitamin E-only groups serve as internal normal values, and the control-group ranges for the serum analytes were consistent with published clinical-chemistry reference intervals for male Wistar rats measured on comparable automated (Cobas) analysers [30,31].

### 3.2. Oxidative Stress (Redox) Parameters

Systemic redox markers did not follow the pattern expected from a purely pro-oxidant exposure. Serum TOS was numerically lower, not higher, in the glyphosate-only groups than in controls (HG 20.19 vs. control 27.99 μmol H_2_O_2_ Eq/L), and OSI likewise decreased across the glyphosate groups (HG 1.40 vs. control 2.69), while TAS rose progressively (HG 1.31 vs. control 1.03 mmol Trolox Eq/L). Thus, at this exposure duration, the circulating oxidant markers did not demonstrate a net systemic increase in oxidant load in glyphosate-exposed animals; this apparent dissociation between serum redox markers and tissue injury is addressed in the Discussion (Section 4.1).

**Figure 1:**
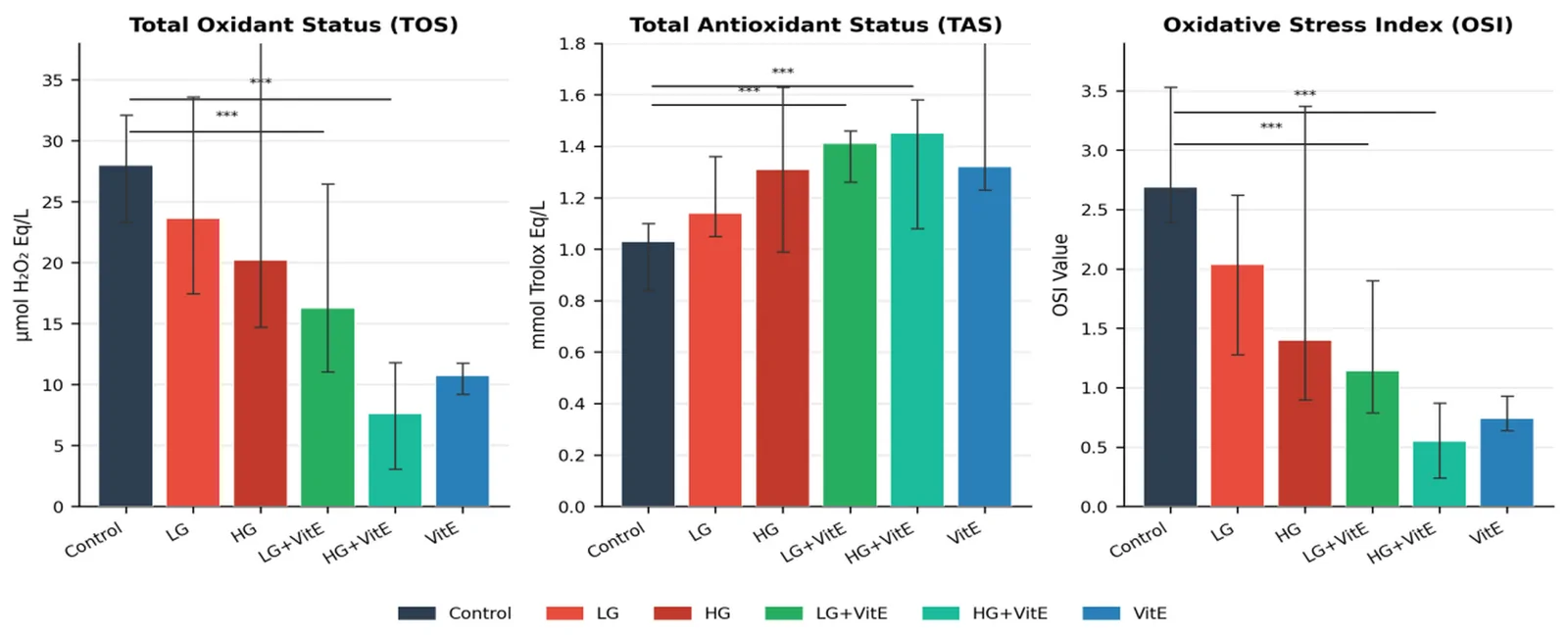
Systemic redox parameters across experimental groups. Bars represent median values; error bars indicate interquartile range. TAS increased and OSI decreased with vitamin E co-treatment; TOS in HG + VitE was significantly lower than in HG (*p* = 0.007). TOS: total oxidant status; TAS: total antioxidant status; OSI: oxidative stress index. *** *p* < 0.001 vs. control.

Against this background, vitamin E co-administration produced a clear antioxidant effect. TAS was significantly enhanced in both vitamin E-treated groups (LG + VitE: 1.41 mmol Trolox Eq/L, *p* < 0.001 vs. control; HG + VitE: 1.45 mmol Trolox Eq/L, *p* = 0.002 vs. control), and OSI reached its lowest values in these groups (HG + VitE 0.55; LG + VitE 1.14). Critically, TOS in the HG + VitE group (7.59 μmol H_2_O_2_ Eq/L) was significantly lower than in the HG group treated with glyphosate alone (*p* = 0.007). These changes are summarised in Figure 1.

### 3.3. Hepatic Function Parameters

**Figure 2:**
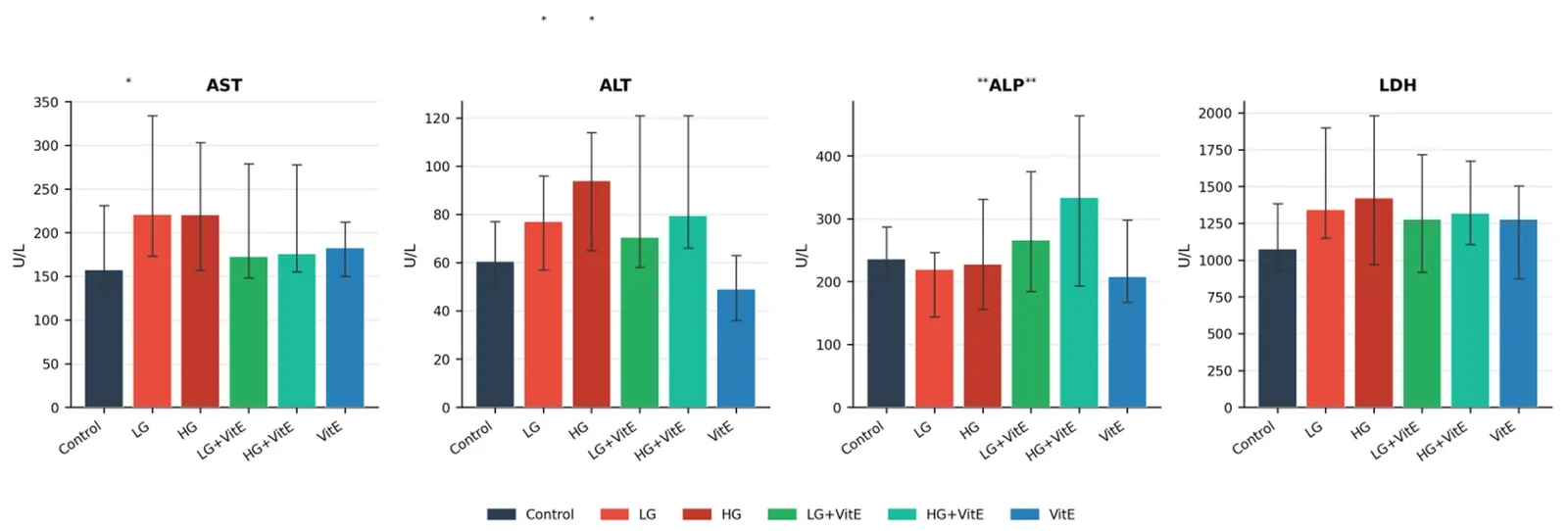
Liver function parameters across experimental groups. Bars represent median values; error bars indicate interquartile range. AST was significantly elevated in the LG group (*p* = 0.045); ALT was elevated in HG and HG + VitE groups (*p* < 0.05); ALP was unexpectedly increased in vitamin E-supplemented groups (*p* < 0.01). * *p* < 0.05; ** *p* < 0.01.

Glyphosate exposure produced significant hepatocellular enzyme changes (Figure 2). AST was significantly elevated in the LG group compared with controls (221 vs. 157.5 U/L; *p* = 0.045). ALT was significantly elevated in the HG group (94 vs. 60.5 U/L; *p* = 0.012) and remained elevated in the HG + VitE group (79.5 U/L; *p* = 0.044 vs. control), indicating only partial hepatoprotection by vitamin E at the higher glyphosate dose. An unexpected and significant elevation in ALP was observed in vitamin E-treated groups (LG + VitE: 266 U/L vs. LG: 219 U/L; *p* = 0.004; HG + VitE: 333.5 U/L vs. HG: 227.5 U/L; *p* = 0.011). The AST/ALT ratio was significantly lower in the LG + VitE group than in the corresponding LG group (2.49 vs. 3.04; *p* < 0.05), consistent with attenuation of the predominantly AST-driven hepatocellular injury seen with glyphosate alone. LDH and total protein levels showed no significant intergroup differences.

### 3.4. Renal Function and Cardiac Biomarker

**Figure 3:**
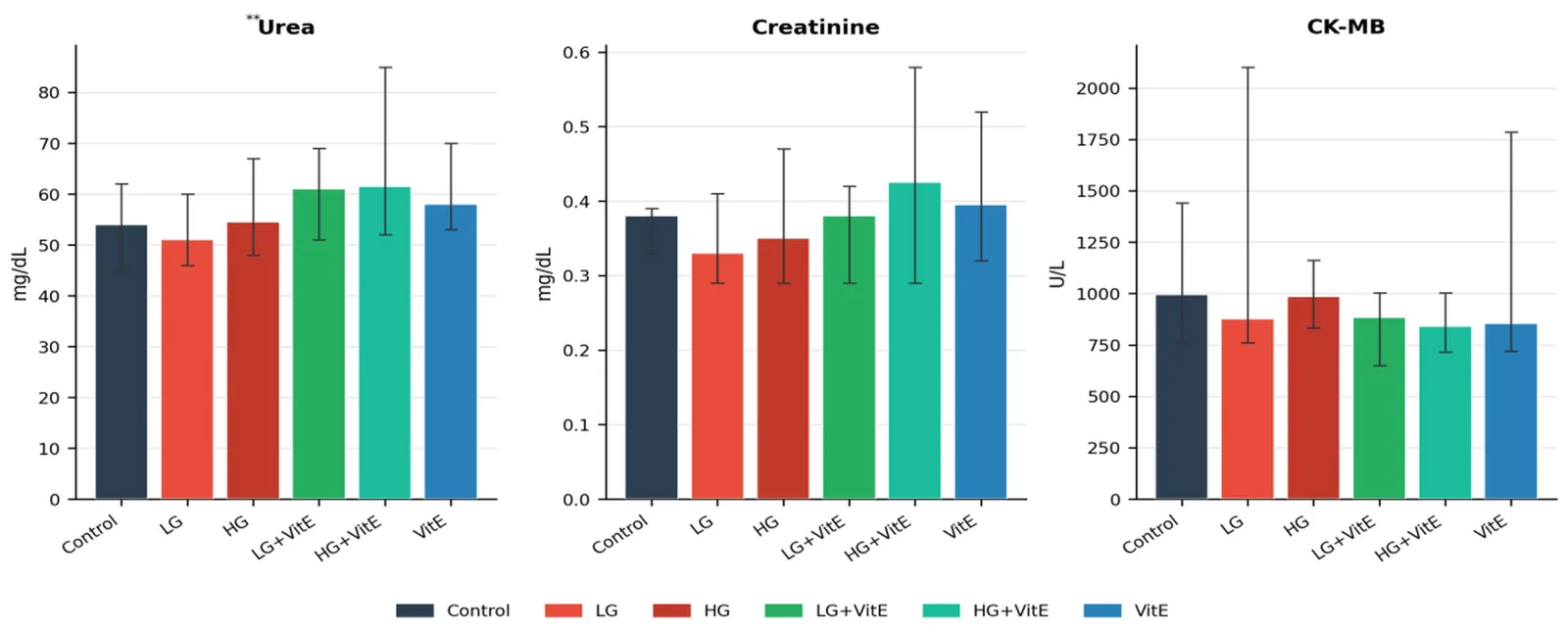
Kidney function markers (urea, creatinine) and cardiac biomarker (CK-MB) across experimental groups. Bars represent median values; error bars indicate interquartile range. Urea was significantly elevated in the LG + VitE group compared with LG (*p* = 0.005). No significant intergroup differences were detected for creatinine or CK-MB. ** *p* < 0.01.

Serum urea was significantly elevated in the LG + VitE group compared with LG (61 vs. 51 mg/dL; *p* = 0.005), but creatinine levels were unaffected across all groups. No significant intergroup differences were observed for CK-MB (Figure 3), indicating the absence of biochemically detectable nephrotoxicity or cardiotoxicity under the tested conditions.

### 3.5. Histopathological Findings

#### 3.5.1. Histopathological Scoring Overview

**Figure 4:**
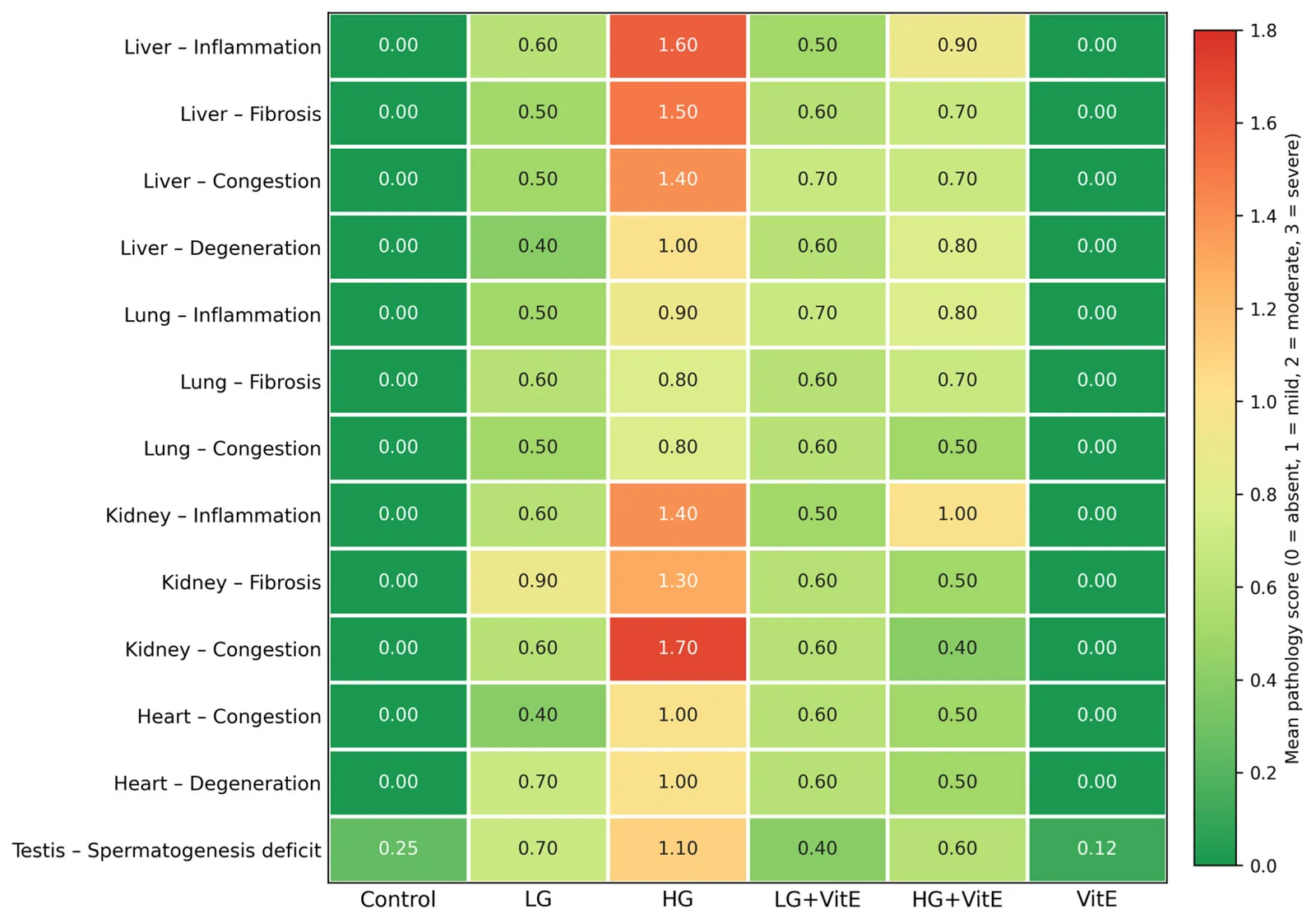
Histopathological scoring heatmap across organ systems and experimental groups, generated from the complete per-animal dataset (Supplementary Table S1). Each cell shows the group mean semi-quantitative score (0 = absent, 1 = mild, 2 = moderate, 3 = severe) for the indicated lesion; the colour scale runs continuously from green (low mean score, ≈absent/mild) through yellow and orange to red (high mean score, ≈moderate/severe). Means are non-integer because they are averaged across animals. High-dose glyphosate (HG) shows the highest scores across organs, and vitamin E co-administration (LG + VitE, HG + VitE) reduces scores across most organ systems. Testis values are a spermatogenesis-deficit proxy calculated as (8 − mean Johnsen score); higher values indicate greater impairment. Scores were generated as described in Section 2.4.

Mean semi-quantitative lesion scores for all organs and treatment groups are summarised in Figure 4, and the complete per-animal scoring data are provided in Supplementary Table S1.

#### 3.5.2. Liver

**Figure 5:**
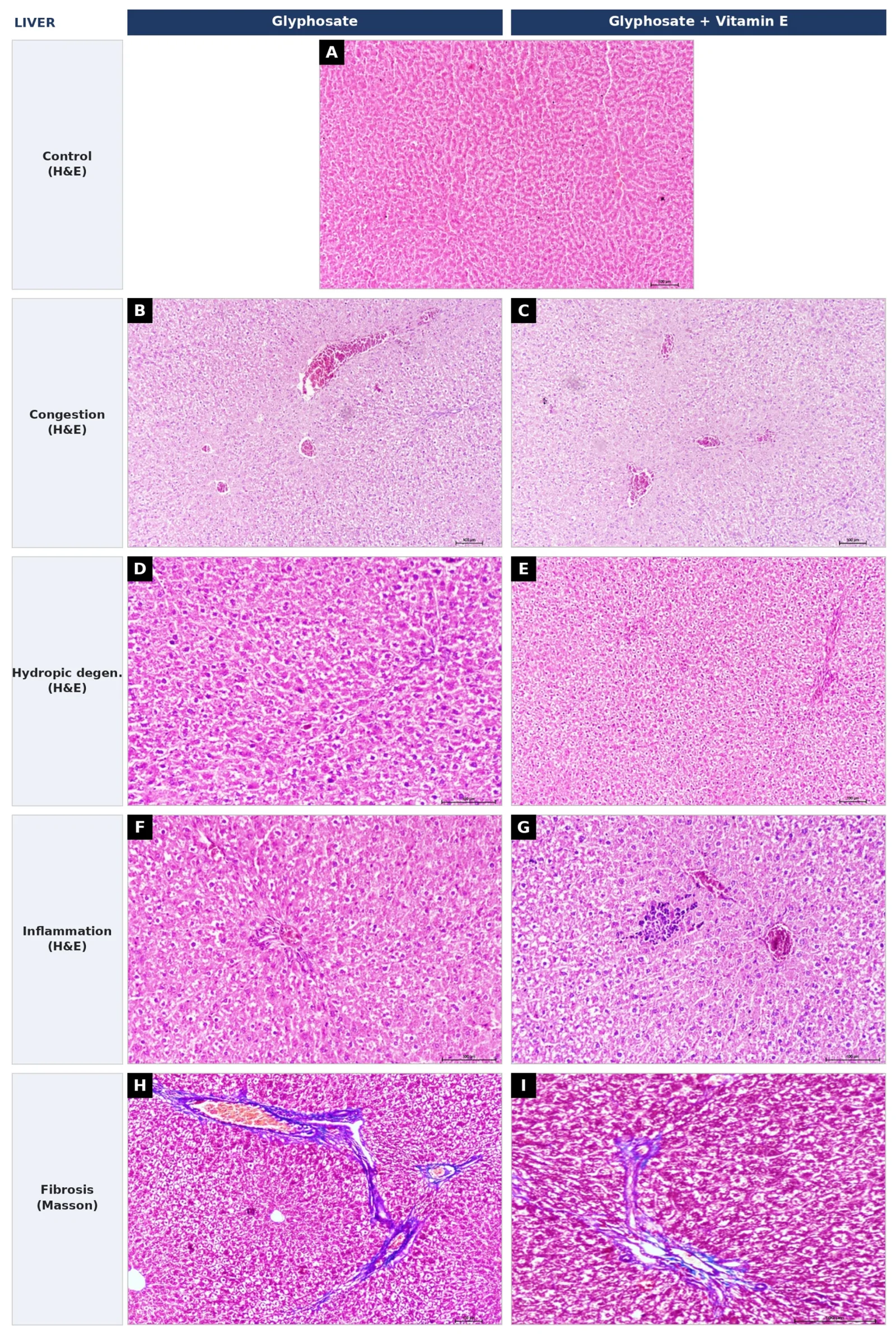
Hepatic histopathological findings. (**A**) Control liver (H&E). (**B**,**C**) Hepatic congestion in the glyphosate (**B**) and glyphosate + vitamin E (**C**) groups, showing sinusoidal dilatation with erythrocyte accumulation (H&E). (**D**,**E**) Hydropic (vacuolar) degeneration in the glyphosate (**D**) and glyphosate + vitamin E (**E**) groups, showing hepatocyte swelling and cytoplasmic vacuolation (H&E). (**F**,**G**) Inflammatory-cell infiltration in the glyphosate (**F**) and glyphosate + vitamin E (**G**) groups (H&E). (**H**,**I**) Hepatic fibrosis in the glyphosate (**H**) and glyphosate + vitamin E (**I**) groups, showing portal-tract collagen deposition stained blue (Masson’s trichrome). In each row the lesion is markedly less severe in the vitamin E-treated group. All panels ×200; scale bar: 100 μm. A single representative control field is shown; a Masson’s trichrome-stained control section was not available (see Limitations).

Compared with the control group, the HG group exhibited the most severe hepatic pathology (Figure 5): sinusoidal dilatation with erythrocyte accumulation (hepatic congestion), hepatocyte swelling with cytoplasmic clearing and vacuolation interpreted on morphology as hydropic (vacuolar) degeneration (special stains for fat and glycogen were not performed to further subtype the vacuoles), and inflammatory-cell infiltration. Inflammation scores were significantly greater in the HG group (mean ± SD: 1.60 ± 0.84) than in the control and VitE groups (*p* = 0.0003; Dunn’s post hoc). Hepatic fibrosis, confirmed by Masson’s trichrome staining as portal-tract collagen deposition (blue), reached the highest score in the HG group (1.50 ± 0.97; *p* = 0.0006; effect size r = 0.805). Vitamin E co-administration consistently reduced all hepatic pathology scores (Figure 5, right column), although differences from the respective glyphosate-only groups did not always reach statistical significance.

#### 3.5.3. Kidney

**Figure 6:**
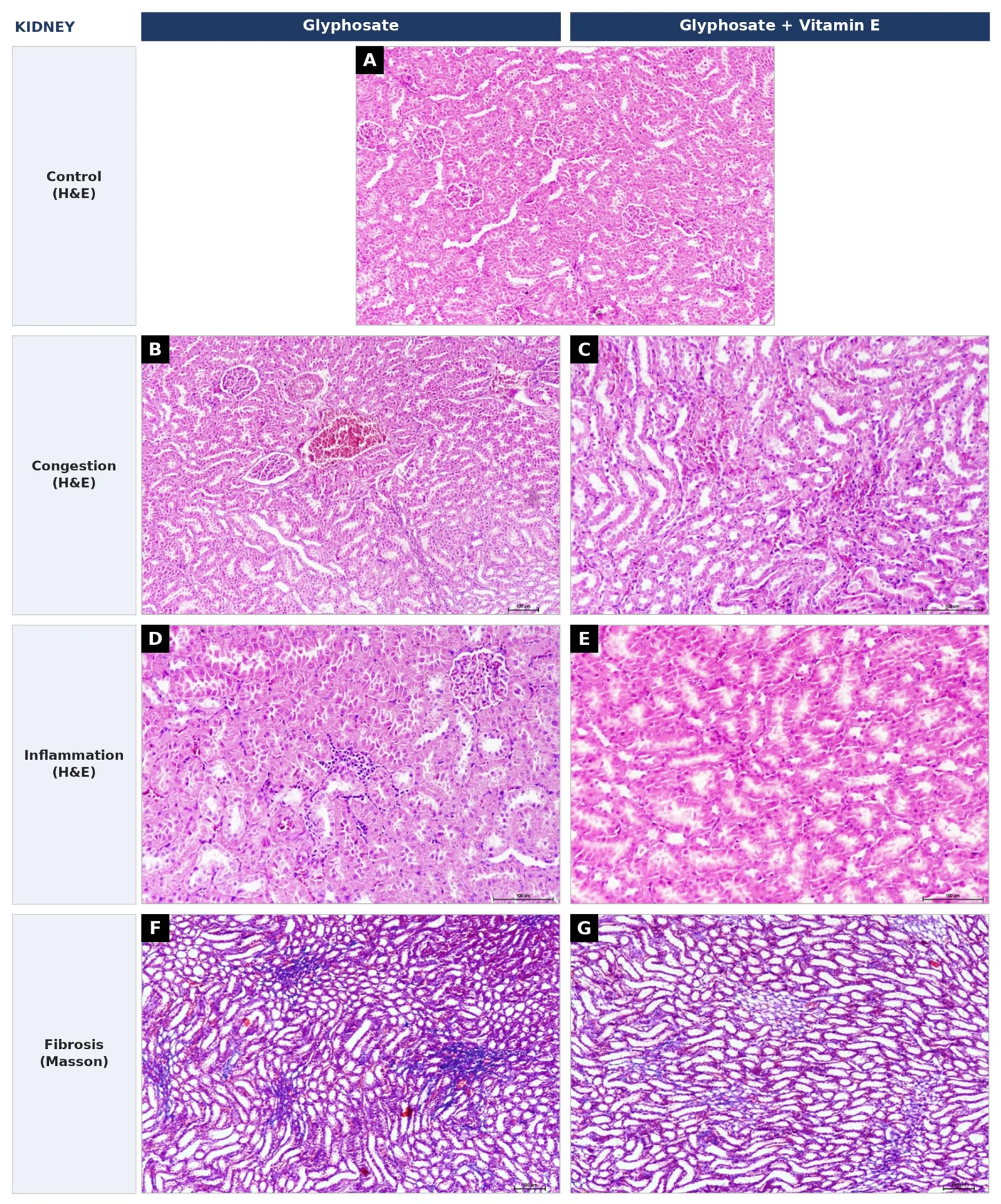
Renal histopathological findings. (**A**) Control kidney (H&E). (**B**,**C**) Renal congestion in the glyphosate (**B**) and glyphosate + vitamin E (**C**) groups, showing glomerular capillary dilatation and peritubular vascular congestion (H&E). (**D**,**E**) Inflammatory-cell infiltration in the glyphosate (**D**) and glyphosate + vitamin E (**E**) groups (H&E). (**F**,**G**) Renal fibrosis in the glyphosate (**F**) and glyphosate + vitamin E (**G**) groups, showing interstitial collagen deposition stained blue (Masson’s trichrome). All panels ×200; scale bar: 100 μm. A single representative control field is shown; a Masson’s trichrome-stained control section was not available (see Limitations).

Renal pathology was characterised primarily by inflammatory, fibrotic and congestion changes (Figure 6). Renal congestion, involving glomerular capillary dilatation with mesangial expansion and peritubular vascular (interstitial capillary) congestion, reached the highest score in the HG group (congestion score: 1.70 ± 0.68; *p* < 0.0001 vs. control; r = 0.888). Renal inflammation was likewise most pronounced in the HG group, and renal fibrosis was confirmed by Masson’s trichrome staining, which demonstrated interstitial collagen deposition (blue) in glyphosate-exposed animals. Renal fibrosis scores were significantly elevated in both glyphosate groups (*p* < 0.05 vs. control), while LG + VitE and HG + VitE groups did not differ significantly from controls, indicating vitamin E protection against fibrotic progression. Renal congestion was significantly reduced in the HG + VitE group compared with HG (0.40 ± 0.52 vs. 1.70 ± 0.68; *p* = 0.007).

#### 3.5.4. Lung

**Figure 7:**
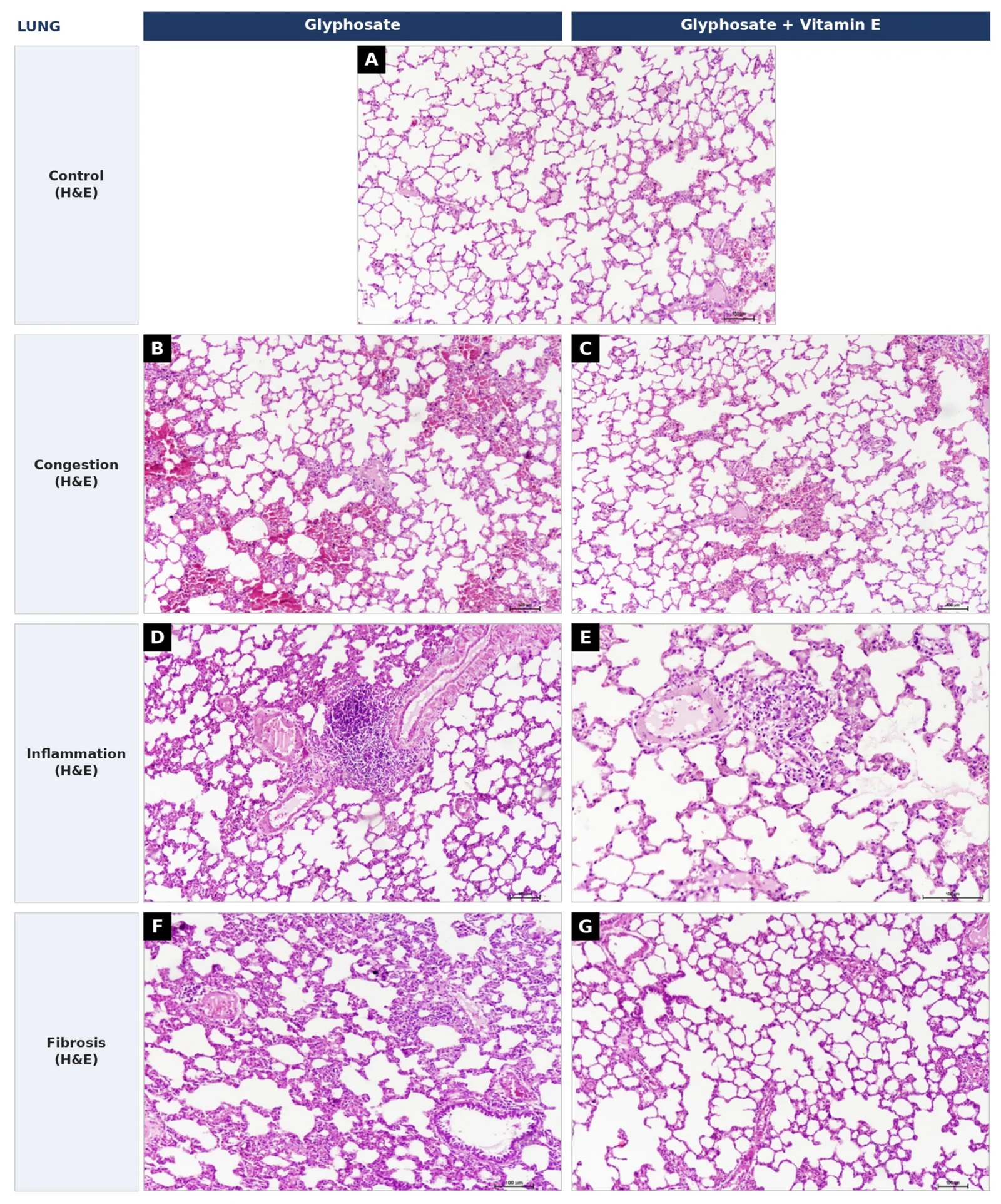
Pulmonary histopathological findings (haematoxylin and eosin). (**A**) Control lung. (**B**,**C**) Pulmonary congestion in the glyphosate (**B**) and glyphosate + vitamin E (**C**) groups, showing capillary engorgement within alveolar septa with reduced alveolar spaces. (**D**,**E**) Mononuclear inflammatory infiltration, predominantly peribronchiolar/perivascular, in the glyphosate (**D**) and glyphosate + vitamin E (**E**) groups. (**F**,**G**) Interstitial thickening in the glyphosate (**F**) and glyphosate + vitamin E (**G**) groups. All panels ×200; scale bar: 100 μm. Collagen-specific staining was not available for the lung, so the fibrosis interpretation is provisional; a single representative control field is shown (see Limitations).

Compared with the control group, pulmonary changes in glyphosate-exposed animals included capillary engorgement within alveolar septa with reduced alveolar spaces (pulmonary congestion) and predominantly peribronchiolar and perivascular mononuclear inflammatory infiltration with some alveolar septal thickening (Figure 7). This peri-airway/perivascular distribution should be interpreted with caution, as it may in part reflect bronchus-associated lymphoid tissue (BALT) rather than a purely diffuse interstitial or intra-alveolar process. Interstitial thickening was also noted; however, because collagen-specific staining was not available for the lung, a definitive diagnosis of pulmonary fibrosis could not be established and this finding is reported only provisionally on H&E. These changes were most marked in the HG group (inflammation: *p* = 0.005; congestion: *p* = 0.011; provisional fibrosis score: *p* = 0.021). Vitamin E treatment reduced the pulmonary inflammation, congestion and provisional fibrosis scores in both treatment groups, although differences from the respective glyphosate-only groups did not reach statistical significance.

#### 3.5.5. Testis

The HG group demonstrated significantly impaired spermatogenesis (Johnsen score: 6.90 ± 0.57) compared with controls (7.75 ± 0.46; *p* = 0.040; r = 0.637) and the VitE group (7.88 ± 0.35; *p* = 0.007). Vitamin E supplementation tended to improve spermatogenesis scores (LG + VitE: 7.60 ± 0.52; HG + VitE: 7.40 ± 0.52), although differences from the respective glyphosate-only groups did not reach statistical significance.

#### 3.5.6. Heart

Cardiac congestion and degeneration were significantly elevated in the HG group compared with controls (mean score: 1.00 ± 0.00 for both; *p* < 0.001; r = 0.960). The LG group also showed significantly elevated cardiac degeneration (0.70 ± 0.48; *p* = 0.004 vs. control). Vitamin E substantially reduced both cardiac congestion (LG + VitE: 0.60 ± 0.52; HG + VitE: 0.50 ± 0.53) and degeneration scores.

## 4. Discussion

This study provides experimental evidence for glyphosate-induced dose-dependent multi-organ histopathological damage and demonstrates the substantial protective efficacy of vitamin E supplementation. The histopathological evidence—spanning liver, lung, kidney, testis and heart—provides morphological confirmation of the hepatocellular-enzyme findings and establishes a pathological basis for glyphosate-induced tissue toxicity.

### 4.1. Redox Balance and the Serum Marker–Tissue Injury Dissociation

A central and initially counter-intuitive observation requires explicit discussion. Although glyphosate produced clear dose-dependent tissue injury, the serum oxidant markers did not indicate a net systemic increase in oxidant load: TOS and OSI were lower in the glyphosate-only groups than in controls, and TAS was higher. Several factors plausibly account for this dissociation. First, circulating TOS/TAS reflect the balance sampled in serum at a single 30-day endpoint and may not capture compartmentalised, tissue-level oxidative injury; local lipid peroxidation in the liver, testis or lung can proceed while systemic markers remain low. Second, the progressive rise in TAS across glyphosate groups is consistent with a compensatory up-regulation of endogenous antioxidant defences in response to a sustained oxidant challenge—an adaptive response frequently reported in subchronic exposure models [32]—which would simultaneously lower measured TOS and OSI. Third, chelation of redox-active transition metals by glyphosate could reduce the metal-catalysed generation of the reactive species that these colorimetric assays detect. Consequently, in this study, the primary evidence for glyphosate toxicity is histopathological rather than serum-redox-based, and the oxidative-stress interpretation is offered as a mechanistic hypothesis supported by the tissue findings rather than proven by the circulating markers. Accordingly, the serum redox data are best interpreted as evidence of the antioxidant action of vitamin E (which lowered TOS below the glyphosate-alone level, HG + VitE vs. HG, *p* = 0.007, and raised TAS) rather than as direct proof that glyphosate raised systemic oxidant load. Direct tissue markers (malondialdehyde, tissue glutathione, ROS-generating enzyme activity) would be required to establish tissue oxidative stress unequivocally and are proposed as the priority next step.

The elevated TAS in vitamin E-treated groups is consistent with the classical antioxidant mechanism: *α*-tocopherol donates a hydrogen atom to peroxyl radicals, generating the relatively stable tocopheroxyl radical, which is subsequently regenerated by ascorbate or glutathione, thereby maintaining antioxidant capacity [20,21]. These composite biomarker findings are consistent with those reported by Klisic et al. [25,33] and support the utility of TOS, TAS and OSI as measures of systemic oxidant/antioxidant balance [26,27].

### 4.2. Hepatotoxicity and Hepatoprotection

The elevation of AST and ALT confirms glyphosate’s hepatotoxic potential [14,15]. Histopathology provided supportive morphological evidence: hepatic congestion with sinusoidal dilatation indicates compromised hepatic microcirculation, likely secondary to endothelial oxidative damage, and hydropic degeneration with hepatocyte vacuolation represents early sublethal cellular injury. Hepatic fibrosis, confirmed by Masson’s trichrome as portal-tract collagen deposition, indicates progression from acute toward chronic hepatic injury; although we avoid over-interpreting this as established cirrhosis, the trichrome-confirmed collagen deposition substantiates a genuine fibrotic response. The concordance between raised transaminases and hepatocellular morphological injury nonetheless provides mutual support for both methodologies.

Vitamin E partially attenuated both biochemical and histopathological hepatic endpoints [22]. The paradoxical elevation of ALP in vitamin E-treated groups warrants careful interpretation. ALP is not liver-specific but is expressed as several tissue isoenzymes, including hepatic/biliary, bone (osteoblast) and intestinal forms [34]. The increase may therefore reflect induction of the hepatobiliary or intestinal isoenzyme, increased bone turnover in these young growing rats, or interaction with the olive oil vehicle through enterohepatic mechanisms rather than hepatotoxicity. Future studies should include ALP isoenzyme fractionation to clarify the tissue source [34].

### 4.3. Pulmonary Manifestations

Pulmonary inflammation, congestion and interstitial change following oral glyphosate administration extend the target-organ profile of this herbicide to respiratory tissue. Following gastrointestinal absorption, glyphosate and its principal metabolite aminomethylphosphonic acid (AMPA) undergo systemic circulation and may reach the pulmonary vasculature, where ROS generation can trigger alveolar-macrophage activation and cytokine-mediated inflammation, consistent with the observed septal thickening. Because this is—to our knowledge—a comparatively under-reported target, and because the observed peri-airway/perivascular distribution of the infiltrate may partly represent bronchus-associated lymphoid tissue and the fibrosis assessment rests on H&E only, we frame the pulmonary interpretation cautiously. Vitamin E consistently reduced pulmonary inflammation, congestion and provisional fibrosis scores, supporting its potential as a protective dietary antioxidant in animals with chronic dietary or environmental glyphosate exposure.

### 4.4. Renal and Reproductive Toxicity

A notable dissociation emerged between renal biochemistry and renal morphology. Serum urea and creatinine remained within the control range across glyphosate groups, yet histopathology revealed glomerular and tubular congestion together with interstitial fibrosis, the latter confirmed by Masson’s trichrome staining, in the high-dose group. This pattern indicates that early structural injury can precede measurable functional decline, since serum creatinine rises only after substantial loss of filtration capacity. The mild elevation of urea in the LG + VitE group, in the absence of any creatinine change, most likely reflects pre-renal or dietary-protein influences rather than intrinsic nephrotoxicity. Renal fibrosis scores were attenuated in both vitamin E co-treated groups, and renal congestion in the HG + VitE group was significantly lower than in HG, supporting a protective effect of vitamin E. The testis showed analogous vulnerability: spermatogenesis, quantified by the Johnsen score, was significantly impaired in the high-dose group. The seminiferous epithelium is rich in polyunsaturated fatty acids and highly susceptible to lipid peroxidation, providing a plausible mechanistic basis for oxidative gonadotoxicity. Vitamin E produced a consistent, though non-significant, trend toward preserved spermatogenesis, in line with its membrane-stabilising antioxidant action.

### 4.5. Relevance to Animal Health and Veterinary Practice

Reframed for a veterinary readership, these findings are most directly relevant to animals with chronic, unavoidable glyphosate intake—for example ruminants grazing recently treated pasture, horses, and farm-kept companion animals—rather than to human occupational exposure. The multi-organ histopathological damage demonstrated here, albeit at doses well above field-realistic intake, supports a precautionary approach to animal exposure and underscores the need for confirmatory studies at lower, environmentally relevant doses and in target species. From a practical standpoint, the robust antioxidant and histoprotective effects of vitamin E suggest that dietary antioxidant supplementation could serve as an adjunctive protective strategy for animals subject to chronic glyphosate exposure [17,35,36], and that feed-additive dosing studies in production and companion animals are warranted.

### 4.6. Proposed Mechanism

**Figure 8:**
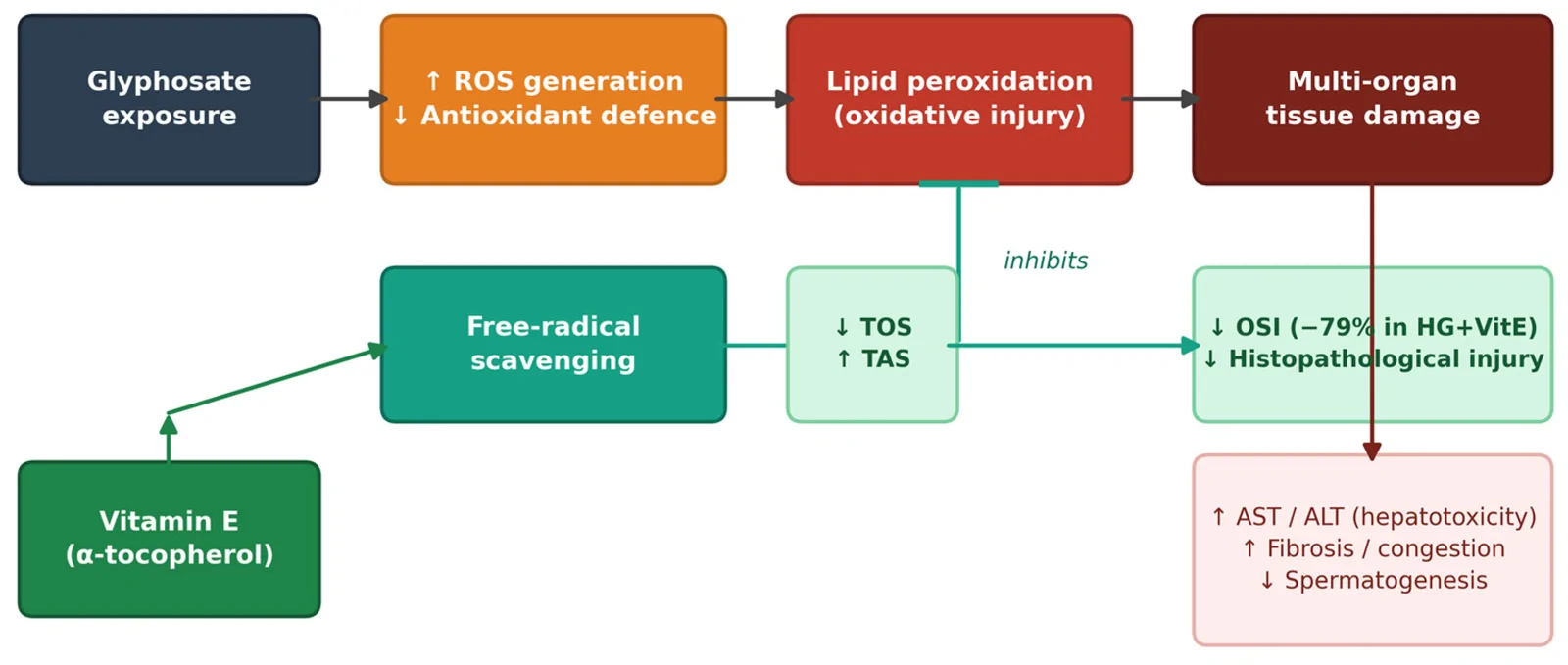
Proposed mechanistic pathway (authors’ hypothesis) illustrating glyphosate-induced multi-organ toxicity and vitamin E-mediated protection. TOS: total oxidant status; TAS: total antioxidant status; OSI: oxidative stress index; ROS: reactive oxygen species.

On the basis of the combined biochemical and histopathological findings, we propose the following interpretive mechanism (Figure 8). Chronic glyphosate exposure drives ROS generation and challenges endogenous antioxidant defences; the resulting lipid peroxidation contributes to the observed multi-organ tissue injury—hepatocellular degeneration and enzyme release, renal and pulmonary congestion, impaired spermatogenesis, and cardiac change. Vitamin E (*α*-tocopherol) is proposed to interrupt this cascade through free-radical scavenging, raising TAS and lowering oxidant load relative to glyphosate alone, and thereby attenuating histopathological injury. This scheme is offered as a hypothesis consistent with the data rather than as a proven pathway, given that the serum markers did not independently confirm a systemic rise in oxidant load (Section 4.1).

### 4.7. Limitations

Several limitations should be acknowledged: (i) a single vitamin E dose was tested—dose-optimisation studies are needed; (ii) only male animals were used, so sex-based differences remain unexplored; (iii) absence of ALP isoenzyme fractionation limits interpretation of the paradoxical ALP elevation; (iv) molecular oxidative-stress endpoints (tissue glutathione, malondialdehyde, ROS-generating enzymes) were not measured, which is particularly relevant given the serum-marker dissociation discussed in Section 4.1; (v) the 30-day exposure may not capture the full spectrum of chronic pathology; (vi) the commercial formulation used (containing 48% glyphosate isopropylamine salt as the declared active ingredient) also includes proprietary surfactant co-formulants that were not disclosed by the manufacturer, so their independent contribution to the observed effects cannot be excluded [18]; (vii) renal and hepatic fibrosis were confirmed by Masson’s trichrome, but pulmonary fibrosis was assessed on H&E only and is therefore reported as provisional pending collagen-specific staining; (viii) although two pathologists scored the sections by consensus, formal quantitative inter-observer reproducibility was not separately reported; and (ix) the histological figures present representative fields only—a single control field per organ was available, a Masson’s trichrome-stained control section and lower-magnification survey (Supplementary) images were not available, and control and lesion fields were not fully magnification-matched, so the spatial distribution of lesions could not be documented and the fibrosis and inflammation interpretations should be regarded as representative rather than exhaustive.

## 5. Conclusions

Chronic exposure to high-dose glyphosate induced dose-dependent multi-organ histopathological damage in male Wistar rats, encompassing hepatic congestion, hydropic degeneration and fibrosis (the last confirmed by Masson’s trichrome); renal congestion, inflammation and trichrome-confirmed interstitial fibrosis; pulmonary inflammation and congestion with provisional (H&E-based) fibrosis; impaired spermatogenesis; and cardiac injury. Although serum oxidant indices did not independently confirm a systemic rise in oxidant load, the tissue findings are consistent with oxidative injury. Vitamin E supplementation (100 mg/kg/day) enhanced systemic antioxidant capacity, lowered oxidant load relative to glyphosate alone, and substantially attenuated histopathological injury across organ systems. These findings support the potential of vitamin E as a protective dietary antioxidant for animals subject to chronic glyphosate exposure and provide a multi-organ morphological evidence base to inform veterinary and food-safety considerations. Confirmatory studies—using pulmonary collagen-specific staining, direct tissue oxidative-stress markers, and lower, environmentally relevant doses in target species—are warranted.

## Figures and Tables

**Table 1:** Experimental groups, glyphosate and vitamin E doses, vehicle, and daily oral gavage volume (30 consecutive days).

Group	*n*	Glyphosate (mg/kg/day)	α-Tocopherol Acetate (mg/kg/day)	Vehicle	Gavage Volume
1. Control	8	—	—	Olive oil	3 mL/kg
2. LG	10	560	—	Olive oil *	3 mL/kg *
3. HG	10	1120	—	Olive oil *	3 mL/kg *
4. LG + VitE	10	560	100	Olive oil	3 mL/kg
5. HG + VitE	10	1120	100	Olive oil	3 mL/kg
6. VitE	8	—	100	Olive oil	3 mL/kg

LG: low-dose glyphosate; HG: high-dose glyphosate; VitE: vitamin E (α-tocopherol acetate). * Glyphosate was diluted in distilled water; olive oil (3 mL/kg) was given to all groups as the tocopherol vehicle, equalising vehicle load and total gavage volume. Doses were selected based on established subchronic toxicological studies [16,23].

**Table 2:** Biochemical and oxidative stress parameters across all experimental groups.

Parameter	Control (*n* = 8)	LG (*n* = 10)	HG (*n* = 10)	LG + VitE (*n* = 10)	HG + VitE (*n* = 10)	VitE (*n* = 8)
**Oxidative Stress Markers**						
TOS (μmol H_2_O_2_ Eq/L)	27.99 (23.35–32.10)	23.63 (17.45–33.61)	20.19 (14.70–38.75)	16.27 (11.04–26.45) ***	7.59 (3.07–11.81) ***##	10.74 (9.20–11.77)
TAS (mmol Trolox Eq/L)	1.03 (0.84–1.10)	1.14 (1.05–1.36)	1.31 (0.99–1.63)	1.41 (1.26–1.46) ***	1.45 (1.08–1.58) **	1.32 (1.23–1.84)
OSI	2.69 (2.39–3.53)	2.04 (1.28–2.62)	1.40 (0.90–3.37)	1.14 (0.79–1.90) ***	0.55 (0.24–0.87) ***	0.74 (0.64–0.93)
**Liver Function Tests**						
AST (U/L)	157.5 (139–231)	221 (173–334) *	220.5 (157–303)	172.5 (148–279)	176 (155–278)	182.5 (150–212)
ALT (U/L)	60.5 (50–77)	77 (57–96)	94 (65–114) *	70.5 (58–121)	79.5 (66–121) *	49 (36–63)
AST/ALT ratio	2.69 (2.31–4.02)	3.04 (2.35–3.48)	2.42 (1.63–3.43)	2.49 (2.14–3.10) #	2.27 (1.51–3.05)	3.54 (2.63–4.86)
LDH (U/L)	1076.5 (928–1384)	1342 (1149–1899)	1420.5 (969–1981)	1277.5 (918–1717)	1319.5 (1105–1672)	1276 (873–1504)
ALP (U/L)	236 (203–287)	219 (144–246)	227.5 (156–331)	266 (184–375) ##	333.5 (193–464) ##	208 (167–298)
Total Protein (g/L)	65.0 (60.2–69.9)	64.3 (59.5–66.0)	66.5 (59.0–72.0)	64.2 (60.2–66.0)	64.9 (55.0–66.5)	63.2 (56.1–69.3)
**Kidney Function Markers**						
Urea (mg/dL)	54 (45–62)	51 (46–60)	54.5 (48–67)	61 (51–69) ##	61.5 (52–85)	58 (53–70)
Creatinine (mg/dL)	0.38 (0.33–0.39)	0.33 (0.29–0.41)	0.35 (0.29–0.47)	0.38 (0.29–0.42)	0.43 (0.29–0.58)	0.40 (0.32–0.52)
**Cardiac Biomarker**						
CK-MB (U/L)	995 (763–1440)	877 (761–2102)	984 (834–1161)	882 (649–1003)	839 (714–1004)	854 (719–1786)

Data presented as median (minimum–maximum). LG: low-dose glyphosate (560 mg/kg); HG: high-dose glyphosate (1120 mg/kg); VitE: vitamin E (100 mg/kg). TOS: total oxidant status; TAS: total antioxidant status; OSI: oxidative stress index; AST: aspartate aminotransferase; ALT: alanine aminotransferase; ALP: alkaline phosphatase; LDH: lactate dehydrogenase; CK-MB: creatine kinase-MB. * *p* < 0.05; ** *p* < 0.01; *** *p* < 0.001 vs. control; # *p* < 0.05; ## *p* < 0.01 vs. respective glyphosate group.

## Data Availability

The original contributions presented in this study are included in the article/Supplementary Material. The complete per-animal histopathological scoring sheet is provided as Supplementary Table S1. Further inquiries can be directed to the corresponding authors.

## Supplementary Materials

The following supporting information can be downloaded at: https://www.mdpi.com/article/10.3390/vetsci13080740/s1, Table S1a: Per-animal semi-quantitative histopathological scores (complete raw dataset, n = 56); Table S1b: Group mean ± SD histopathological scores (calculated from S1a)
